# Protective effects of extracts from *Acer truncatum* leaves on SLS-induced HaCaT cells

**DOI:** 10.3389/fphar.2023.1068849

**Published:** 2023-03-15

**Authors:** Yanxiao Fan, Ronghui Gu, Ruifei Zhang, Miaomiao Wang, Heran Xu, Min Wang, Chunlin Long

**Affiliations:** ^1^ Key Laboratory of Ecology and Environment in Minority Areas (Minzu University of China), National Ethnic Affairs Commission, Beijing, China; ^2^ College of Life and Environmental Sciences, Minzu University of China, Beijing, China; ^3^ Key Laboratory of Plant Resource Conservation and Germplasm Innovation in Mountainous Region (Guizhou University), Ministry of Education, Guiyang, China; ^4^ School of Liquor and Food Engineering, Guizhou University, Guiyang, China; ^5^ College of Chemistry and Materials Engineering, Beijing Technology and Business University, Beijing, China; ^6^ BTBU-TANGYI Innovation Center for the Evaluation of the Safety and Efficacy of Bioengineering Raw Materials, Beijing, China; ^7^ Key Laboratory of Ethnomedicine (Minzu University of China), Ministry of Education, Beijing, China; ^8^ Institute of National Security Studies, Minzu University of China, Beijing, China

**Keywords:** *A. truncatum*, dermatitis, interleukin 6, flavonoids, skin inflammations

## Abstract

**Introduction:** *A. truncatum* Bunge (Sapindaceae or formerly Aceraceae) is a tall deciduous tree native to China. Traditionally, the leaves of *A. truncatum* are decocted and used by Chinese Mongolians, Koreans, and Tibetans to treat skin itching, dry cracks, and other skin ailments, which indicates *A. truncatum* leaves may have a potential inhibitory effect on various skin inflammations.

**Methods:** To examine the protective effect against skin inflammations of *A. truncatum* leaf extract (ATLE), an *in vitro* dermatitis model was established using sodium dodecyl sulfate (SLS)-induced HaCaT cells. The anti-inflammatory effect of ATLE was evaluated by analyzing cell viability, apoptosis, reactive oxygen species (ROS), interleukin 6 (IL-6), and prostaglandin E2 (PGE2) levels.

**Results:** Orthogonal experiments showed that the pretreatment with ATLE can reduce the IL-6 levels, PGE2 levels, and apoptosis increased in SLS-stimulated HaCaT cells, which indicates that ATLE has positive efficacy for dermatitis. Furthermore, three flavonoid compounds kaempferol-3-*O*-*α*-L-rhamnoside, quercetin-3-*O*-*α*-L-rhamnopyranoside, kaempferol-3,7-di-*O*-*α*-L-rhamnoside, and 1,2,3,4,6-Penta-*O*-galloyl-*β*-D-glucopyranose (PGG) were isolated and identified. Among them, kaempferol-3,7-di-*O*-*α*-L-rhamnoside was isolated from this plant for the first time. These compounds have been proven to have an anti-inflammatory effect. They may contribute to the efficacy of *A. truncatumin* treating skin inflammation.

**Discussion:** The results revealed that ATLE has the potential to be used as an additive in various skin care products to prevent skin inflammations and may be incorporated in formulations for topical application as a therapeutic approach against dermatitis.

## 1 Introduction

Dermatitis is a chronic skin inflammatory disease characterized by severe itching with recurring and prolonged episodes (Wüthrich, 1999; Hoffjan and Epplen, 2005; Bussmann et al., 2007; Stemmler and Hoffjan, 2016). Both internal and (or) external factors can cause dermatitis by destroying the skin epidermis (Rowlands et al., 2006; Fujii et al., 2015). Internal factors are usually genetic defects of filaggrin FLG, chronic digestive system diseases, mental stress, insomnia, excessive fatigue, emotional changes, endocrine disorders, infections, metabolic disorders, etc. External factors include sunlight, cold, dryness, heat, animal fur, plants, cosmetics, soap, artificial fibers, food, and climate change (Barker et al., 2007; Fuxench, 2020; Ruge et al., 2020). The pathogenesis of dermatitis remains unclear at present. Abnormal immune system function has been widely considered a primary cause (Klein et al., 2008; Hidaka et al., 2016; Eigenmann, 2019).

As a relapsed and refractory disease without a thoroughly investigated pathogenesis, dermatitis’s treatment can be toilsome. Hormone drugs and antihistamines are commonly used to treat dermatitis clinically (Meltzer et al., 2020; Tanei, 2020). However, both have side effects and are unacceptable for some patients (Tan et al., 2013; Mcgregor et al., 2015; Kim and Kim, 2016). Compared with western medicine, various traditional medicinal systems such as traditional Chinese medicine (TCM) system have the characteristics of long-term practice, small side effects and fundamentally conditioning and curing diseases (Wong et al., 2006; Thorburn et al., 2013). Consequently, it has become a research hotspot to find reliable drugs for treating atopic skin diseases like dermatitis and acne from traditional medicines (Kamei et al., 2021; Kildaci et al., 2021; Nakatsuji et al., 2021).

Keratinocytes account for about 95% of human epidermal cells. After the skin is infected by bacteria and fungi, resulting in inflammation, keratinocytes will respond and then manifest the symptoms on the surface of the skin (Girolomoni and Pastore, 2001; Miodovnik et al., 2012). Sodium Lauryl Sulfate (SLS), also known as Sodium dodecyl sulfate (SDS), is a surfactant with a strong cleaning ability and strong degreasing ability (Aguiar et al., 2011; Ade-Browne et al., 2019). It is also one of the most irritating surfactant components and is widely used in cleanser, shampoo, and shower gel. However, due to its strong degreasing power, it is easy to cause damage to sensitive skin (Wu and Hettiarachchy, 1998; Gabard et al., 2010; Petersen et al., 2010; Stettler et al., 2021). SLS is also a common positive control for human skin irritation model patch tests.

SLS can destroy the barrier function of the stratum corneum. SLS stimulation can induce epidermal inflammatory cell infiltration, neutrophils migrate to the skin epidermis, and induce skin inflammation (Wilhelm et al., 1994; Charbonnier et al., 2001; Kim et al., 2015; Polat et al., 2015; Tavares et al., 2020). When the skin surface is stimulated by chemical substances, the release of the pro-inflammatory factor IL-6 in keratinocytes increases, and the increased expression of IL-6 can promote the release of PGE2, and promote the expression of TNF and cause a local inflammatory response, manifested as edema, erythema, itching (Peritt et al., 1992; Fiebich et al., 2010; Hsu et al., 2015). Recent studies have shown that keratinocytes can generate danger signals under the stimulation of sensitizers and activate the autoimmune system through the activation of TLR (Martin et al., 2011; Zhao et al., 2016). When MAPK and NF-κB signaling pathways were activated through signal transduction, the expression of pro-inflammatory mediators would be induced (Mogensen, 2019). Further, the Kyoto Encyclopedia of Genes and Genomes (KEGG) database, which was used to predict diseases that differentially expressed genes may cause, showed that keratinocytes stimulated by SLS activated a series of expressions of genes related to immunity and skin inflammation. The diseases that may be incurred are atopic dermatitis and allergic and autoimmune diseases (Zhao et al., 2016).

*A. truncatum* Bunge (Sapindaceae or formerly Aceraceae) is an ethnomedicinal plant native to China. It is naturally distributed in Inner Mongolia, Jilin, Liaoning, Gansu, and other areas in China (Wu et al., 2010). According to previous ethnobotanical studies, different linguistic groups, including Mongolians, Tibetan, Koreans, and Han Chinese in northern China, have collected the leaves of *A. truncatum* to treat itching, wounds, and other diseases for centuries. For example, some Mongolian people in the Inner Mongolia Autonomous Region of China will pick fresh *A. truncatum* leaves in summer and autumn and make them into tea. Long-term drinking the tea can prevent hypertension and hyperlipidemia. Also, some Korean people in Changbai County, Baishan City, Jilin Province, China will directly boil fresh *A. truncatum* leaves and drink them or wash the wounds to eliminate internal heat and inflammation (Gu, 2019).

Relevant literature about *A. truncatum* were searched in Google Scholar, Sci Finder, Web of Science, Scopus, Springer Link, PubMed, Wiley, China National Knowledge Infrastructure (CNKI), Baidu Scholar, and China Science and Technology Journal Database to made literature quality evaluation. The results showed that there were 289 articles related to the traditional use, chemical composition, pharmacological activity, and safety of *A. truncatum*. The earliest available documents were published in 1949, while the latest one was published in February 2023. However, through the summary of the research on *A. truncatum,* we found that previous studies on this plant mainly focused on the extraction and isolation of chemical components, and their biological activities. No research attempted to explore the potential of *A. truncatum* in the treatment of skin diseases or the application in skin-caring (Gu et al., 2019a; Gu et al., 2019b; Fan et al., 2021; Zhang et al., 2022).

The previous phytochemical studies showed that the main chemical components of *A. truncatum* were polyphenols, organic acids, or esters (Zhang et al., 2008; Tan et al., 2016; He et al., 2019; Fan et al., 2021; Leoty-Okombi et al., 2021; Song et al., 2021). Our group speculates that the *A. truncatum* leaf extract (ATLE) has a therapeutic effect on skin inflammation caused by various external stimuli, and this therapeutic effect may be related to its main chemical components. Based on our hypothesis, we established a skin inflammation model in HaCaT cells treated with SLS in this study. To determine the role of ATLE in treating skin inflammation, the PGE2 and IL-6 level properties of ATLE in the SLS-induced HaCaT cells dermatitis model were evaluated. In addition, the main chemical components in the ATLE to verify were extracted and separated to enable a chemical characterization of the tested extract. The aim of this study is to investigate the efficiency of extracts from *A. truncatum* leaves through a skin inflammation model in HaCaT cells treated with SLS, and to explore the potential to use it for skin care in the future.

## 2 Material and methods

### 2.1 Chemicals and instruments

SLS (catalog No: L4509), Fetal bovine serum (FBS, catalog No: F8687), Phosphate Buffered Saline (PBS, catalog No: BSS-1006), Dulbecco’s modified Eagle’s medium (DMEM, catalog No: D0819), EDTA-trypsin (catalog No: 59428C), and Penicillin-Streptomycin Solution (PSS No: TMS-AB2) were obtained from Sigma-Aldrich (St. Louis, Missouri, the United States).

Analytical balance (AL204, Mettler Toledo Co., Ltd., Switzerland), ultrasonic cleaner (KQ-5200E, Shufeng Enterprise Co., Ltd., China.), rotary evaporator (BUCHI R-210, Buchi Co., Ltd., Switzerland), vacuum diaphragm pump (LVS301zp, Ilmvac Co., Ltd., Germany), high-speed tissue grinder (KZ-II, Wuhan Seville Biotechnology Co., Ltd., China), ultra-pure water system (milli-r012plus, Millipore Co., Ltd., the United States), Nuclear magnetic resonance spectrometer (Bruker Avance 500 MHz, Bruker Co., Ltd., German), high performance liquid chromatograph (Waters 2695, Waters Co., Ltd., the United States), circulating preparation high performance liquid chromatograph (LC-9110NEXT, JAI Co., Ltd., Japan), high-speed counter-current chromatography (TBE-20A; TBE-300B, Shanghai Tongtian Co., Ltd., China), mass spectrometer (Ltq Orbitrap Discovery, Thermo Fisher Scientific Co., Ltd., the United States), Flex PCR instrument (Quant StudioTM 6, Thermo Scientific Technology Co., Ltd., the United States), microscope (leica DM4B, Leica Instruments Co., Ltd., Germany), constant temperature incubator (WH-05, Wiggens Co., Ltd., Germany), centrifuge (Corning, Sigma-Aldrich Co., Ltd., the United States), cellometer (K2, Nexcelom Co., Ltd., the United States), multifunctional microplate reader (Envision, PerkinElmer Co., Ltd., United States), and flow cytometer (EXFLOW-206, Dakowi Co., Ltd., China) were used in the experiment.

### 2.2 Preparation of ATLE

The *A. truncatum* leaves were collected from Fenghuangling Mountain in Beijing, identified by Professor Chunlin Long at the Minzu University of China. The voucher specimens were deposited in the Herbarium of Minzu University of China.

1.2 kg of air-dried *A. truncatum* leaves were crushed and passed through a 40-mesh sieve. The sieved powder was extracted with 12 L 95% ethanol at room temperature, 2 days per time, stirring 10 min for every 5 h. The extraction was repeated three times. The extract was filtered and combined. The combined filtrate was concentrated under reduced pressure with a rotary evaporator and dried at 40°C to obtain 425 g of the total extract.

### 2.3 Cell culture

Human immortalized keratinocytes cells (HaCaT cells) were used to evaluate the anti-inflammatory activity of ATLE. Such cells can form a barrier on human skin to prevent damage caused by heat, ultraviolet radiation, water loss, pathogenic bacteria, fungi, parasites, and viruses. When the skin is stimulated by adverse factors from the outside, they are the first to respond.

HaCaT cells provided by the Cell Bank of the Chinese Academy of Sciences, were cultured in a 25 cm^2^ cell culture flask (catalog No: CLS430372, Sigma-Aldrich, the United States) in a constant temperature incubator (37°C, 5% CO_2_) for 24 h, with DMEM containing 1% PSS and 10% FBS as the culture medium. The culture medium was replaced after 24 h to remove non-adherent cells and was changed every 1 to 2 days until the cells grew and converged. When the cell adhesion reached 70% to 80%, the cells were digested with 0.25% EDTA-trypsin digestion solution and passaged.

### 2.4 Viability assay

After counting with a cell meter, HaCaT cells (8 × 10^3^ cells/well) were seeded in 96-well plates (catalog No: CLS3922, Sigma-Aldrich, the United States) with DMEM containing 10% FBS and 1% PSS at 37°C in a humid 5% CO_2_ atmosphere and treated with 10, 4, 2, 1.5, 1, 0.5, 0.1 mg/mL ATLE for 24 h. DMEM blank medium was used as the blank control group.

ATLE was dissolved with DMEM blank medium and assisted by ultrasonic cleaner (the frequency was 20–50 kHz, the power was 50–500 W, and the temperature should not exceed 40°C), an appropriate proportion of dimethyl sulfoxide (DMSO) was used to help dissolve. After dissolution, use polytetrafluoroethylene membrane (pore diameter is 0.45/0.22 μm) filter the solution to remove impurities. The same method was used to prepare SLS solution.

After 24 h, 10 μL of Cell Counting Kit-8 (CCK-8, catalog No: 96992, Sigma-Aldrich, the United States) CCK8 solution added to each well and place it in an incubator for 2 h. A multifunctional microplate reader was used to detect the optical density (OD) at 450 nm wavelength to calculate the cell survival rate. The formula is survival rate = **(**OD Drug—OD Blank)/(OD Control—OD Blank) × 100%, the concentration of the model when screening LD_50_. The same method was used to determine the concentration of the stimulus SLS.

### 2.5 Model establishment

SLS can destroy the barrier function of the stratum corneum and is a positive substance commonly used in skin irritation evaluation. Studies have shown that SLS stimulation mainly conducts signal transduction through the TNF signaling pathway, which can cause the expression of factors including IL-6, PGE2, TNF, CCL5, CCL20, CXCL8, CXCL3, CSF2, CSF1, TNFAIP3, and NLRC in HaCaT cells, thereby triggering dermatitis and activating the immune system (Zhao et al., 2016).

The HaCaT cells were taken in the logarithmic growth phase, inoculated evenly in a 96-well plate according to the amount of 8 000 cells per well, and cultured in a 37°C, 5% CO_2_ incubator for 24 h. Then, the SLS powder and ATLE was dissolved with blank DMEM, appropriate concentration SLS (0.1 mg/mL) and ATLE (0.5 mg/mL) were used to treat HaCaT cells for 24 h after the cells adhered to the wall.

According to the order of adding stimulator SLS and drug ALTE, we tested different experimental models. The protective model was pre-processing, which involved adding drug ATLE first to protect HaCaT cells, and then stimulated the cells with SLS. The synchronous model was simultaneous processing, this model required add ATLE and SLS in HaCaT cells simultaneously. The therapeutic model was post-processing, that is, HaCaT cells were stimulated with SLS and then treated with ATLE. The HaCaT cells viability was detected and the appropriate experimental model was determined.

### 2.6 Cellular ROS detection and apoptosis analysis

Total ROS production in HaCaT cells was determined by the 2′,7′-dichlorodi-hydrofluorescein diacetate (DCFH-DA) fluorescence method (catalog No: MAK143-1KT, Sigma-Aldrich, the United States). DCFH-DA was diluted at 1:1000 with a serum-free medium to a final concentration of 10 μM/L. After the cells were collected, they were suspended in diluted DCFH-DA and incubated in a 37°C cell incubator for 20 min Invert and mix every 3 to 5 min to ensure adequate contact between the probe and the cells. Cells were washed three times with a serum-free cell culture medium to sufficiently remove DCFH-DA that did not enter the cells. The fluorescence intensity, before and after stimulation, was detected under the wavelengths 488 and 525 nm, respectively. SLS-induced HaCaT cell apoptosis was analyzed by flow cytometer. Appropriately treated HaCaT cells were harvested and washed in cold PBS, centrifuged, and re-suspended in an annexin-binding solution to which a working solution of FITC-annexin V and PI was added. After 15 min incubation at 25°C, the samples were immediately analyzed under single laser emitting excitation by a flow cytometer.

### 2.7 Anti-inflammatory mechanism of ATLE

SDS stimulates HaCaT cells to cause overexpression of pro-inflammatory factor TNF, activates inflammatory cytokines and immune-related chemokines through the TNF signal pathway, triggers the inflammatory response, and activates and recruits leukocytes to inflammatory sites to play an immune function. Finally, it regulates cell proliferation, differentiation, and apoptosis. In this process, inflammatory cytokines IL-6 and PGE2 play a key role.

To reveal the anti-inflammatory mechanism of ATLE, HaCaT cells were pretreated with or without 0.5 mg/mL ATLE and then treated with serum-free medium containing 0.2, 0.1, 0.08, 0.06, and 0.04 mg/mL SLS for 24 h. Meanwhile, the HaCaT cells were stimulated by 0.1 mg/mL SLS after pretreated with or without serum-free medium containing 10, 4, 2, 1.5, 1, 0.5, and 0.1 mg/mL ATLE for 24 h as a control. The supernatant medium was collected from each well and centrifuged at 1000 r/min for 5 min to remove cell debris. The IL-6 and PGE2 levels of it were detected by the ELISA kit (catalog No: RAB0313, Sigma-Aldrich, the United States; catalog No: E4637-100, BioVision, the United States).

### 2.8 Separation and purification of chemical components of ATLE

In order to determine the main chemical constituents of *A. truncatum* leaves, 420 g ATLE was dissolved in water. Ethanol was added as little as possible per time during the stirring and dissolving process to increase the solubility, and the final concentration of ethanol was less than 10%. The sample solution was added to the pre-activated D101 macroporous resin column with 1 g sample: 10 g macroporous resin (catalog No: S14161-500g, Shanghai Yuanye Biotechnology Co., Ltd, China), and then the gradient elution was carried out with pure water, 20%, 40%, 60%, 80% and 95% ethanol solution in sequence. Six fractions A-F were obtained. The sample of faction C was separated with a polyamide column (200–300 mesh), and the gradient elution was carried out with methanol-water solution (v/v l:1; 2:1; 4:1) to obtain C_l_-C_3_ components.

**FIGURE 1:**
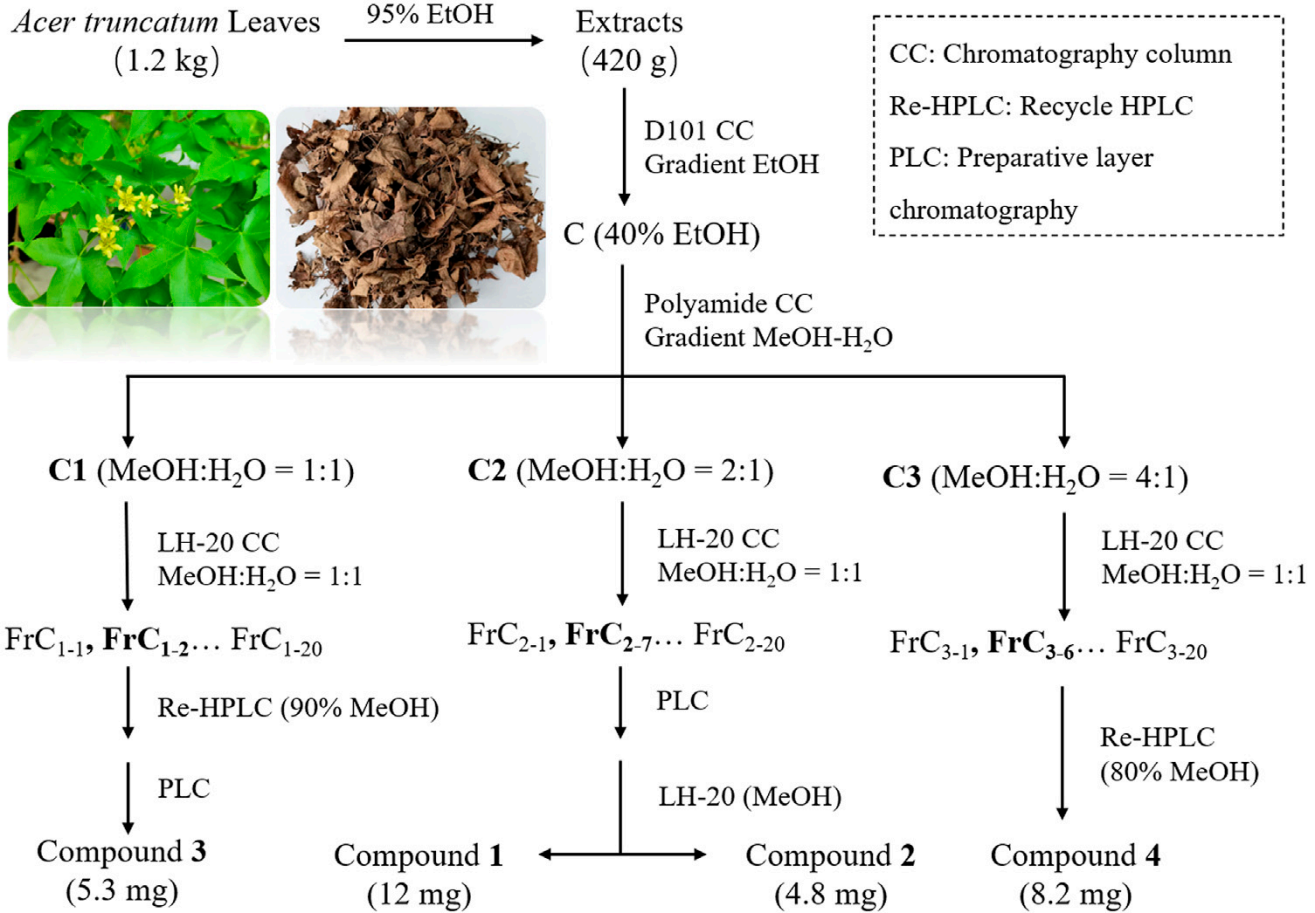
Schematic diagram of compound 1-4 isolation.

Faction C_2_ was further separated and eluted by gel Sephadex LH-20 (methanol: water 1:1) to obtain fractions FrC_2-1_—FrC_2-20_. Frc_2-7_ was prepared by thin-layer silica gel plate scraper, and then purified and washed by gel Sephadex LH-20 in solvent methanol. Then, compound **1** and compound **2** were obtained, respectively. Faction C_1_ was separated and eluted with a solvent (methanol: water 1:1) using gel SephadexLH-20 to obtain fraction FrC_1-1_—FrC_1-20_. The main point obtained after the separation of FrC_1-2_ by cyclic preparation of high-performance liquid chromatography (HPLC) (90% methanol) continued to be purified by the scraper to obtain compound **3**. In addition, the fraction C_3_ was separated and eluted with gel Sephadex LH-20 (methanol: water 1:1) to obtain fractions FrC_3-1_—FrC_3-20_. FrC_3-6_ was separated by cyclic preparation of HPLC (80% methanol) to obtain compound **4**. The workflow of compound 1–4 is shown in Figure 1.

### 2.9 Statistical analysis

All experimental results have been repeatedly verified at least three times. All statistical results are the mean value ± standard deviation of the experimental results. SPSS software was used for the one-way ANOVA test of experimental data. If *p* < 0.05, the results were significantly different.

## 3 Results

### 3.1 SLS and ATLE on HaCaT cells

**FIGURE 2:**
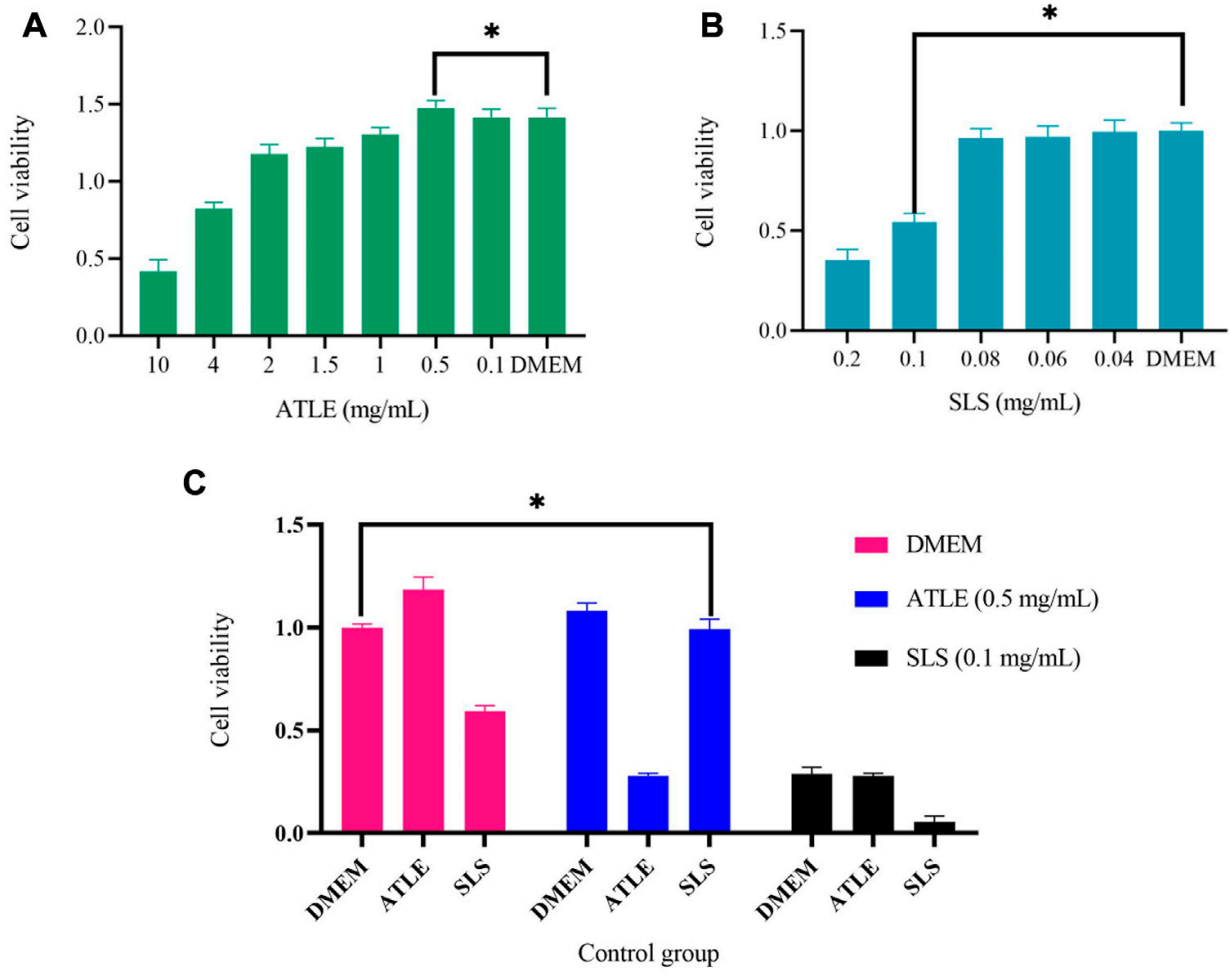
The effects of Sodium Lauryl Sulfate (SLS) and *A. truncatum* leaf extract (ATLE) on HaCaT cells. Cell viability was analyzed by Cell Counting Kit-8. **(A)** The HaCaT cells were treated with 10–0.1 mg/mL ATLE for 24 h **(B)** HaCaT cells were treated with 0.2–0.04 mg/mL SLS for 24 h **(C)** HaCaT cells were pre-treated with 0.5 mg/mL ATLE and 0.1 mg/mL SLS for 24 h with DMEM used as a negative control. *p < 0.05.

Cell viability was analyzed by Cell Counting Kit-8 (CCK-8). As shown in Figure 2, The survival rate of HaCaT cells was 54.18% under 0.1 mg/mL SLS for 24 h of incubation, which indicated that 0.1 mg/mL could be the threshold of HaCaT tolerance SLS. Excessive SLS dose will lead to high cell mortality, and if the dose is too small, the stimulation to cells will not be obvious enough. The results of HaCaT cells treated with ATLE are shown in Figure 2B. When cells were treated with 0.5 mg/mL ATLE, the cell viability was 104.28%, which exceeded the viability of cells treated with negative control DMEM, and the cell viability of HaCaT cells increased in a dose-dependent manner.

0.5 mg/mL ATLE, and 0.1 mg/mL SLS were used for pre-treatment cells for 24 h, and DMEM was used as a negative control, then the cells were treated with 0.5 mg/mL ATLE, 0.1 mg/mL SLS and DMEM for 24 h successively, the results are shown in Figure 2C, which points out that pretreatment with 0.5 mg/mL ATLE can more effectively increase the cell viability.

### 3.2 Effect of ATLE on ROS and survival rate

**FIGURE 3:**
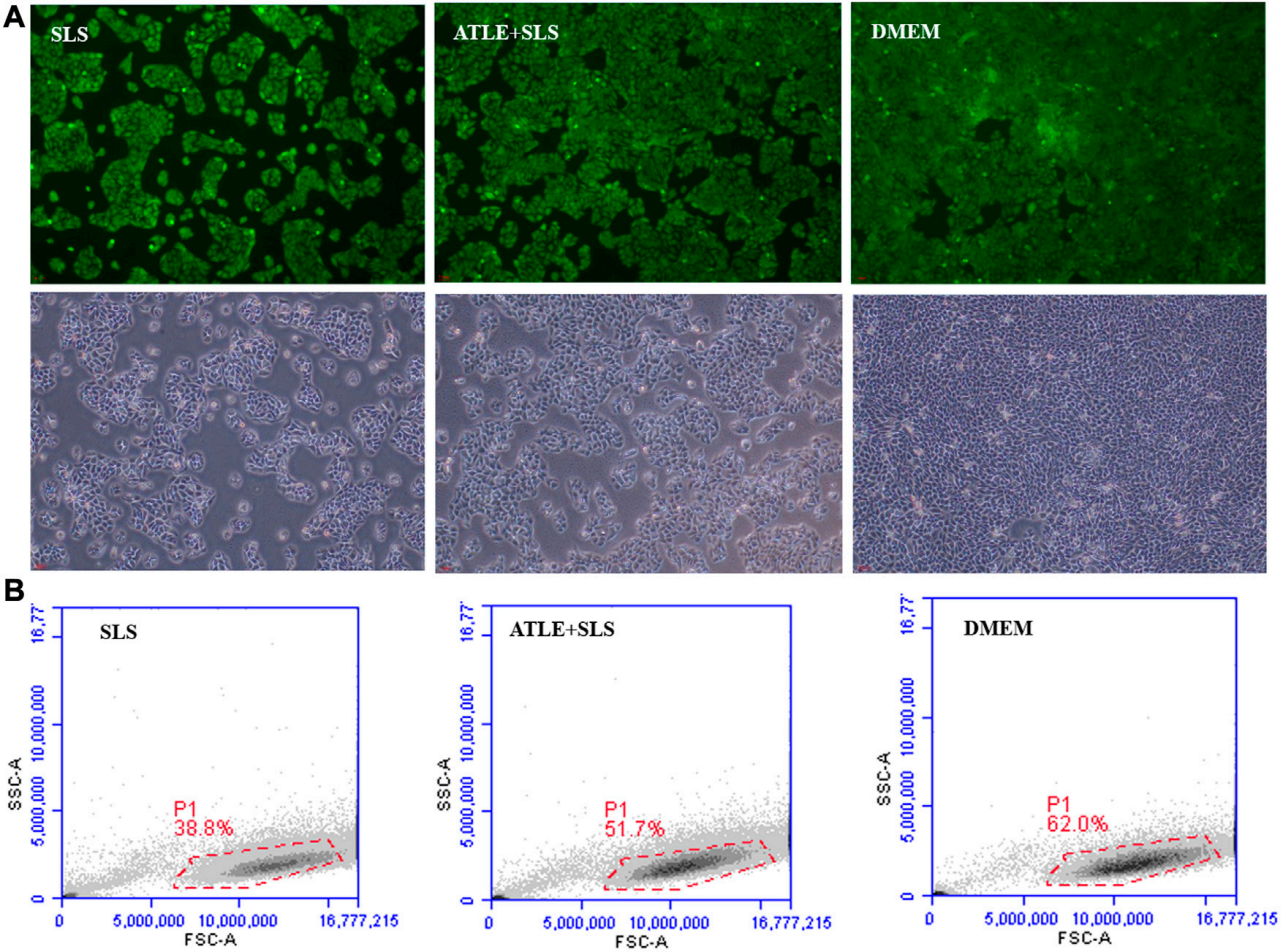
Inhibition of ATLE on ROS and apoptosis. **(A)** The intracellular ROS was detected with DCF-DA staining. **(B)** To investigate the effect of ATLE on cell apoptosis, the survival rate of HaCaT cells was analyzed by flow cytometry. *p < 0.05.

Inflammation leads to oxidative stress, which induces apoptosis. In order to further explore the effect of ATLS on ROS and apoptosis induced by SLS, the intracellular ROS was detected by fluorescence quantitative method, and the apoptosis rate was detected by flow cytometry, to evaluate the antioxidant activity of ATLE. The results are shown in Figure 3. Green fluorescence in HaCaT cells indicated intracellular ROS localization. When HaCaT cells were treated with SLS, the fluorescence intensity was higher than that in the control group (Figure 3A), and the cell survival rate in specific areas was only 38.8% (Figure 3B), indicating that SLS led to HaCaT cells inflammation. After pretreatment with ATLE, the survival rate of HaCaT cells increased to 51.7%, which was close to 62% of DMEM in the control group, indicating that ATLE has a certain inhibitory effect on cell apoptosis induced by SLS (*p* < 0.05).

### 3.3 ATLE downregulates the expression of PGE2 and IL-6

The role of Prostaglandin E2 (PGE2) in the field of inflammation is very diverse. In the past few decades, studies using COX-2, m-PGES1, and EP receptor gene knockout mice have made new and important findings, proving that prostaglandins have both pro-inflammatory and anti-inflammatory effects. These effects are usually produced by targeted regulation of gene expression in related tissues (Kvirkvelia et al., 2010; Yu et al., 2017). PGE2 is usually an essential pro-inflammatory mediator. All the main signs involved in inflammation are edema, redness, swelling, pain, and itching. In addition, Interleukin 6 (IL-6) is an important indicator of inflammation. The increase of IL-6 level indicates that there is inflammation in the body. The more obvious the increase of IL-6, the more serious the inflammation in the body (Neurath and Finotto, 2011; Akar-Ghibril et al., 2020). Therefore, the expression level of inflammatory cytokines PGE2 and IL-6 in supernatant of SLS, 0.5 mg/mL ATLE-SLS, ATLE, and ATLE-0.1 mg/mL SLS treated HaCaT cells was measured by an Elisa kit.

**FIGURE 4:**
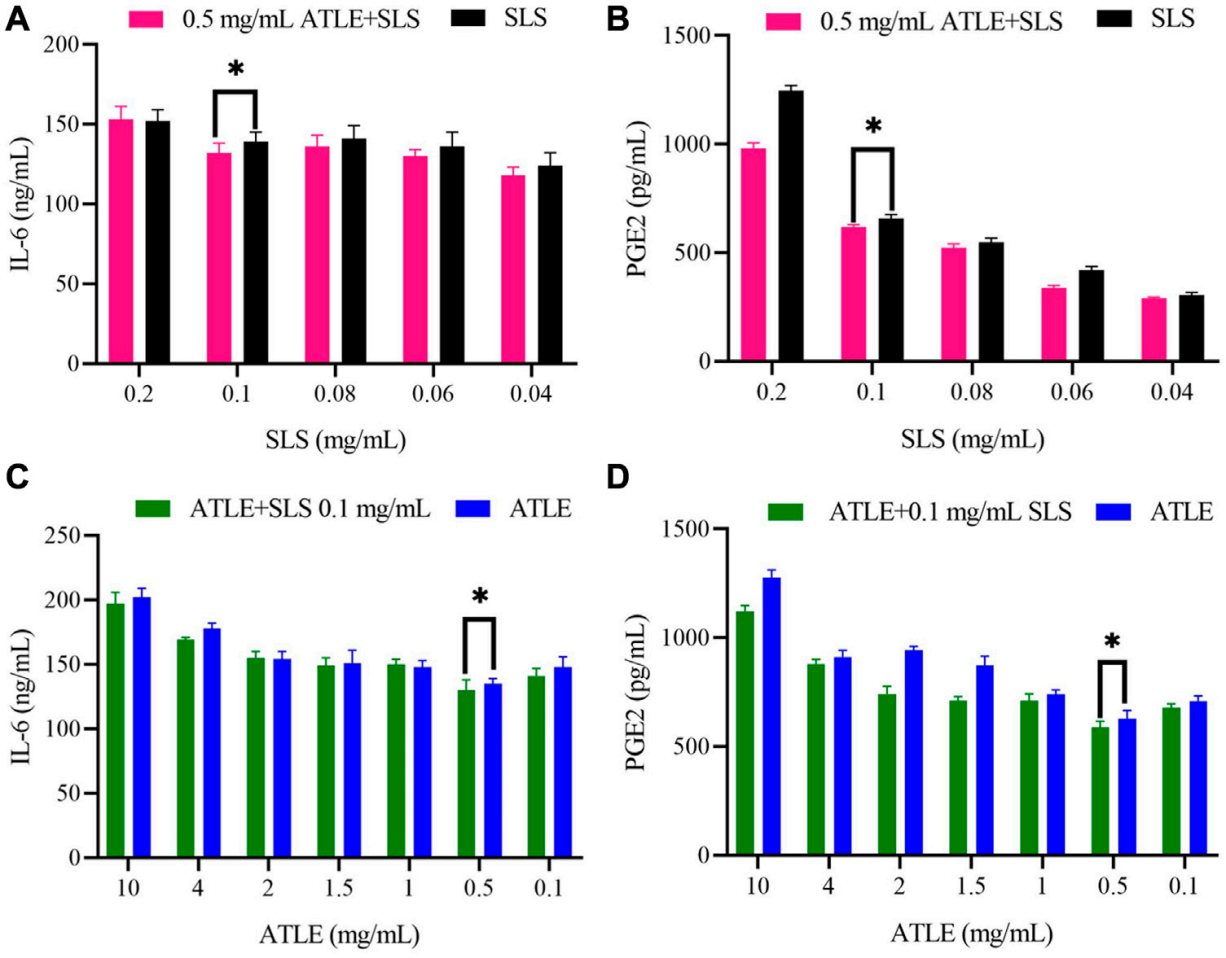
Interleukin 6 (IL-6) and prostaglandin E2 (PGE2) level in supernatant of SLS, 0.5 mg/mL ATLE-SLS, ATLE, and ATLE-0.1 mg/mL SLS treated HaCaT cells. **(A)** The released IL-6 levels in only the SLS group and 0.5 mg/mL ATLE-SLS group were measured with IL-6 Elisa Kit. **(B)** The released PGE2 levels in only the SLS group and 0.5 mg/mL ATLE-SLS group were measured with the PGE2 Assay Kit. **(C)** The released IL-6 levels in only the ATLE group and ATLE-0.1 mg/mL SLS group were measured with IL-6 Elisa Kit. **(D)** The released PGE2 levels in only the ATLE group and ATLE-0.1 mg/mL SLS group were measured with the PGE2 Assay Kit. *p < 0.05.

It can be found in Figure 4 that although there are fluctuations, the expression levels of IL-6 and PGE2 in 0.5 mg/mL ATLE + SLS group HaCaT cells were significantly lower than those in SLS stimulated group. The expression levels of IL-6 and PGE2 showed a concentration dependent relationship with SLS. With the decrease of SLS concentration, the expression levels of IL-6 and PGE2 also showed a downward trend, but the level of PGE2 decreased more significantly than that of IL-6, indicating that PGE2 plays a vital role in HaCaT cells inflammation caused by SLS (Figures 4A, B). Meanwhile, the levels of IL-6 and PGE2 in ATLE-0.1 mg/mL SLS group were generally lower than those in ATLE group. The levels of IL-6 and PGE2 basically decreased with the decrease of ATLE concentration (Figures 4C, D). The results above are consistent with the detection results of CCK-8 (Figures 2A, B).

The research results shows that ATLE pretreatment could inhibit the inflammation caused by SLS stimulating HaCaT cells. By down regulating the expression of inflammatory cytokines PGE2 and IL-6, ATLE reduces the adverse stimulation of HaCaT cells from SLS and plays a protective role on HaCaT cells.

### 3.4 Structure identification of compounds isolated from ATLE

**FIGURE 5:**
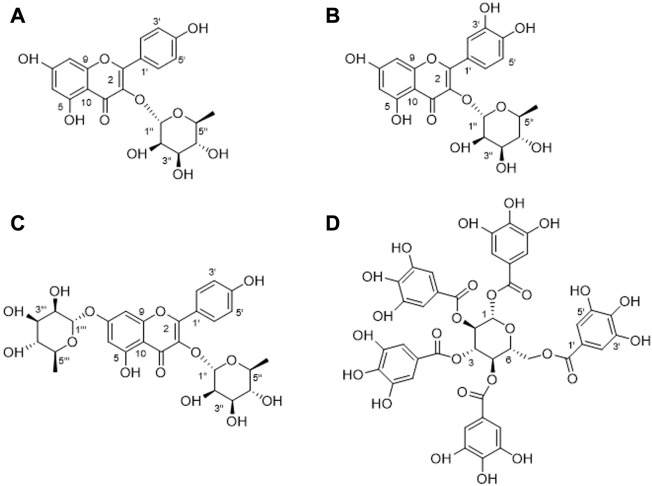
Structures of four compounds isolated from *A. truncatum* leaves. **(A)** Kaempferol-3-*O*-*α*-L-rhamnoside. **(B)** Quercetin-3-*O*-*α*-L-rhamnopyranoside. **(C)** Kaempferol-3,7- di -*O*-*α*-L-rhamnoside. **(D)** 1,2,3,4,6-Penta-*O*-galloyl-*β*-D-glucopyranose.

Using conventional column chromatography such as D101 macroporous resin, silica gel, Sephadex LH-20 gel, and polyamide resin, combined with the preparation of thin-layer chromatography and cyclic preparation of HPLC, four compounds were isolated from *A. truncatum* leaves. The structures were identified by nuclear magnetic resonance and high-resolution mass spectroscopical data, through comparing to those data reported in the literature. Results showed that these compounds were identified as Compound **1**: kaempferol-3-*O-α-L-*rhamnoside (Figure 5A), Compound **2**: quercetin-3-*O*-*α*-L-rhamnopyranoside (Figure 5B), Compound **3**: kaempferol-3,7-di-*O*-*α*-L-rhamnoside (Figure 5C), and Compound **4**: 1,2,3,4,6-penta-*O*-galloyl-*β*-D-glucopyranose (Figure 5D). The amount of the four compounds is 12, 4.8, 5.3, and 8.2 mg. Their yields are 10.91%, 4.36%, 6.63%, and 7.32%, respectively.

#### 3.4.1 Compound **1**

Yellow powder, HR-ESI-MS: *m*/*z* 433.2154 [M + H]^+^, the molecular formula is C_21_H_20_O_10_. ^1^H NMR spectrum showed 12 aromatic carbons, suggesting that the compound contains two benzene rings. According to the coupling constants of these aromatic hydrogens, they are a tetrasubstituted benzene ring *δ* 6.26 (d, *J* = 2.0 Hz, 1H), 6.11 (d, *J* = 2.0 Hz, 1H), and an AABB disubstituted benzene ring *δ* 7.75 (d, *J* = 8.7 Hz, 2H), 6.93 (d, *J* = 8.8 Hz, 2H), respectively. An anomeric proton at *δ* 5.37 (d, *J* = 1.7 Hz, 1H) and methyl at *δ* 0.93 (m, 3H) indicated the presence of a rhamnose moiety in 1. In the ^13^C NMR spectrum, *δ* 179.97 suggested a carbonyl group, and *δ* 17.80 suggested the presence of a methyl group. In addition, *δ* 131.94 and 116.74 both showed strong resonances, suggesting there are two pairs of overlapped resonances. Compared to the reported NMR data, compound **1** was identified as kaempferol-3- *O*-*α*-L-rhamnoside (Fang and Ye, 2008).

#### 3.4.2 Compound **2**

Yellow powder, the protonated ion *m/z* 449.2314 [M + H]^+^ in HR-ESI-MS spectrum indicated the molecular formula C_22_H_24_O_10_. Five aromatic protons can be observed in the ^1^H NMR spectrum, combined with the coupling constants of these aromatic hydrogens. It can be found that there are two benzene rings in compound 2, which are a trisubstituted benzene ring *δ* 7.33 (d, *J* = 2.0 Hz, 1H), 7.29 (dd, *J* = 8.2, 2.0 Hz, 1H), 6.91 (d, *J* = 8.2 Hz, 1H) and a tetra-substituted benzene ring *δ* 6.36 (d, *J* = 2.0 Hz, 1H), 6.19 (d, *J* = 1.7 Hz, 1H). Additionally, an anomeric proton at *δ* 5.35 (d, *J* = 1.5 Hz, 1H) and a methyl at *δ* 0.94 (d, *J* = 5.9 Hz, 3H) suggest the presence of a rhamnose group in this compound. In the ^13^C NMR spectrum, *δ* 179.74 suggested a carbonyl group, and *δ* 17.78 suggested the presence of a methyl group. By comparing with the NMR data in the literature, compound 2 was identified as quercetin-3-*O*-*α*-L-rhamnopyranoside (Fossen et al., 1999).

#### 3.4.3 Compound **3**

Yellow powder. The molecular formula of C_27_H_30_O_14_ was predicted by the molecular ion *m/z* 579.338 [M + H]^+^ in the HR-ESI-MS spectrum. Similarly, there are six aromatic protons in the ^1^H NMR spectrum. Combined with the molecular formula of the compound, it can be inferred that the structure has two substituted benzene rings. Two anomeric protons at *δ* 5.56 (d, *J* = 3.4 Hz, 1H), 5.40 (d, *J* = 1.6 Hz, 1H), and two methyls at *δ* 1.26 and 0.94 suggested that there are also two rhamnoses in **3**. In the ^13^C NMR spectrum, *δ* 179.97 suggested a carbonyl group, 18.22 and 17.83 suggested the presence of two methyl groups. In addition, there are two pairs of overlapped resonances at δ 132.16 and δ 116.87, suggesting that compound **3** is a di-glycoside of flavonoid. The compound is identified as kaempferol-3,7-di-*O*-*α*-L-rhamnoside by comparing with the reported ^1^H and ^13^C NMR data (Wang et al., 2017).

#### 3.4.4 Compound **4**

White powder. The negative ion peak of the compound is *m/z* 939.0056 [M – H]^–^, suggesting that its molecular formula is C_41_H_32_O_26_. In the ^1^H NMR spectrum, there are 10 singlet protons in the low-field aromatic region. Their peak intensities appear as five sets of highly overlapped aromatic hydrogens: *δ* 7.11 (2H, s), 7.05 (2H, s), 6 .98 (2H, s), 6.95 (2H, s) and 6.90 (2H, s), it is speculated that there are five tetrasubstituted benzene rings in this compound. A set of glucose at *δ* 93.96, 74.57, 74.25, 72.34, 69.95, and 63.26 were suggested in the ^13^C NMR spectrum, combined with five ester carbonyl groups: [*δ* 168.07, 167.44, 167.16, 167.07, 166.37] and 30 overlapped aromatic carbons. Presumably, there are five galloyl groups in this compound. Through a comprehensive analysis of ^1^H and ^13^C NMR data and a comparison with related literature, it is determined as 1,2,3,4,6-penta-*O*-galloyl-*β*-D-glucopyranose (Cho et al., 2010).

## 4 Discussion

### 4.1 Development potential of daily chemical products associated with *A. truncatum*

Sodium Lauryl Sulfate (SLS) is a surfactant that frequently encountered in daily life. It is widely used in textiles, food, medicine, cosmetics, and other fields. It is often functioned as a foaming agent for facial cleanser, toothpaste gel, and shampoo (Petersen et al., 2010; Stettler et al., 2021). However, excessive use of SLS will also cause a series of side effects, especially skin mucosal damage and allergic reactions, which are manifested as severe itching, dry peeling, and redness of the skin. Therefore, it is critical to find potential natural drugs to treat skin inflammation caused by excessive use of daily chemicals containing SLS (Wang et al., 2010; Tang et al., 2019; Liu et al., 2021). Many research examples show that ethnomedicine and its related traditional knowledge are vital clues and foundations for us to find new drugs, which are significant for solving some serious diseases that affect human wellbeing (Jin et al., 2021; Natella et al., 2021; Chaachouay et al., 2022; Chen et al., 2022).

In the nomadic areas in northern China, *A. truncatum* leaves are traditionally used to treat skin itching and wounds. However, due to a lack of scientific verification and improvement of medical conditions, this little-known traditional medicinal practice is on the verge of disappearing. To restore the traditional medicinal practice of *A. truncatum* leaves, SLS was selected as a stimulator to construct an inflammatory model of HaCaT cells. ATLE was then used in anti-inflammation experiments. The result has confirmed that ATLE can protect HaCaT cells from damage caused by SLS stimulation and reduce cell apoptosis (Figure 3). Our research shows that ATLE has an outstanding anti-inflammatory effect, and the traditional medicinal use of *A. truncatum* is of scientific significance.

**FIGURE 6:**
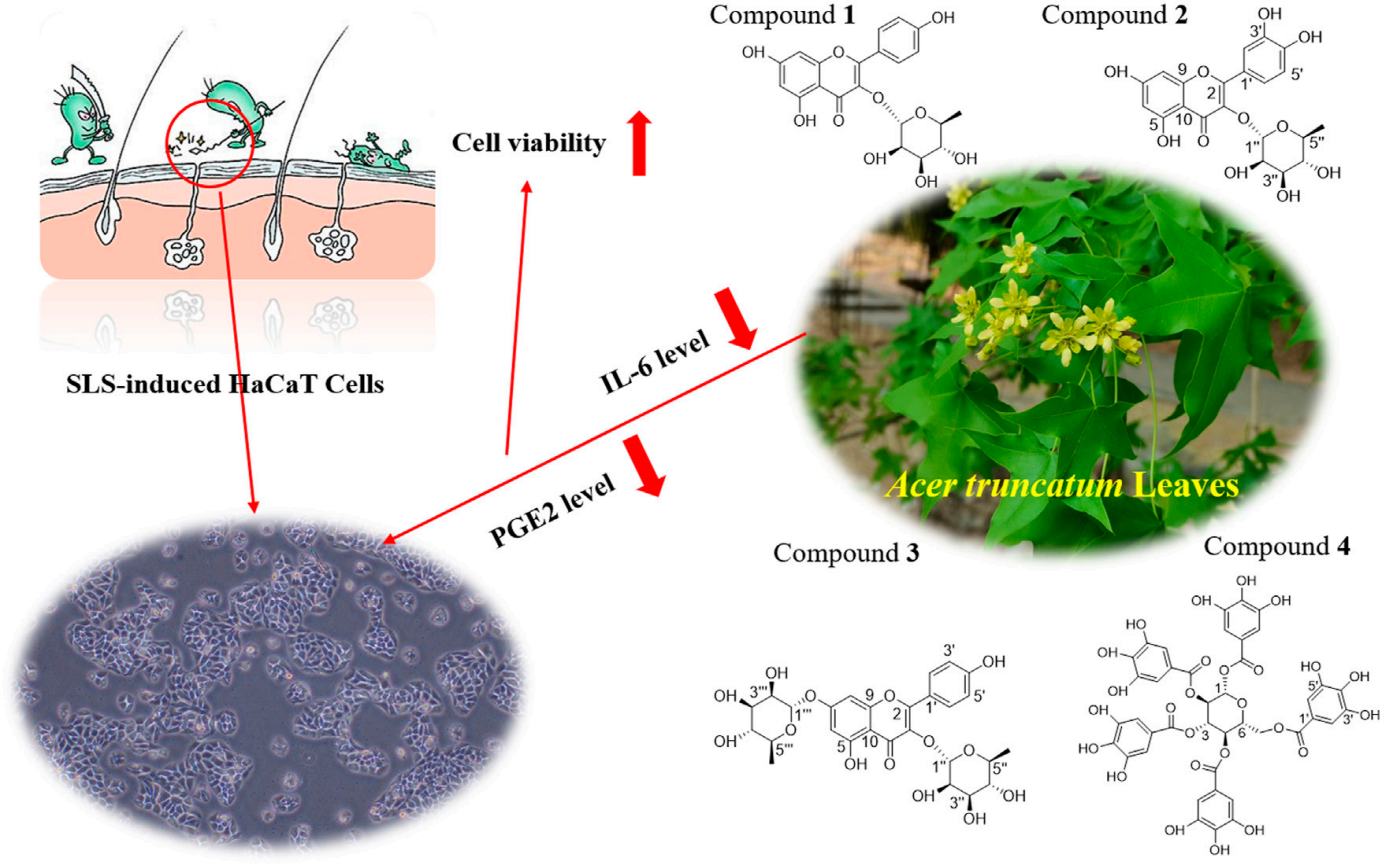
Underlying potential mechanism of the anti-inflammatory effect of *A. truncatum* leaves. The anti-inflammatory effect of *A. truncatum* leaves might be associated with downregulation of IL-6 and PGE2. Four flavonoids from *A. truncatum* leaves may contribute to its anti-inflammatory activity.

IL-6 and PGE2 are proinflammatory factors and are directly related to inflammation (Lin et al., 2010; Kawada et al., 2015; Tsai et al., 2018; Ahsan et al., 2021). IL-6 is a multi-effect inflammatory factor that affects the inflammatory reaction. The increase of IL-6 usually indicates an inflammatory reaction in the body. In the process of infection, trauma, surgery, stress reaction, tumor generation, and other acute inflammatory reactions, IL-6 will be rapidly generated. The level of IL-6 is closely related to inflammation, viral infection, and other autoimmune diseases. In the case of systemic infection, the increase of IL-6 will be more obvious (Artaza-Irigaray et al., 2019; Malashenkova et al., 2021; Prairie et al., 2021). PGE2 is the primary inflammatory mediator of inflammatory diseases such as rheumatoid arthritis, osteoarthritis, hormone dermatitis, and allergic dermatitis. PGE2 can induce inflammation, promote local vasodilation, increase capillary permeability, and cause symptoms such as redness, swelling, pain, and heat. In addition, PGE2 has been shown to inhibit Th1 differentiation, B cell function, T cell activation, and allergic reaction. When inflammation occurs, the expression of PGE2 in natural immune cells such as neutrophils, monocytes, and natural killer cells will be significantly increased to play its anti-inflammatory role (Sreeramkumar et al., 2012; Nasrallah et al., 2016; Elwakeel et al., 2019; Stojanovska et al., 2022). By comparing the expression levels of IL-6 and PGE2 in HaCaT cells, we found that the levels of IL-6 and PGE2 produced by SLS stimulation in HaCaT cells were decreased by ATLE, which indicates that *A. truncatum* leaves have a therapeutic effect on skin inflammation caused by SLS (Figure 6). This research is the first to reveal that *A. truncatum* has significant anti-inflammatory activity, which provides strong evidence for the traditional use of *A. truncatum* leaves to treat itchy skin and heal skin wounds. This is also a clue for the development of a daily skincare product based on *A. truncatum* leaves extract. Future research can focus on the possibility of *A. truncatum* leaves extract in daily chemical products, such as common face creams, repair lotions, body milk, toner, etc.

### 4.2 Scientific exposition of *A. truncatum* related traditional knowledge

Flavonoids exist widely in plants and are one of the most important metabolic components for plants to resist external environmental pressure. Due to their broad-spectrum biological activities, they have also been widely used in human healthcare (Gu, 2019; Gu et al., 2019b; Fan et al., 2021). In this study, the chemical constituents of *A. truncatum* leaves were extracted and separated by a variety of chromatographic techniques. The structures of the isolated compounds were identified by nuclear magnetic resonance spectroscopy, high-resolution mass spectrometry, and literature data. Four compounds were preliminarily isolated and identified from *A. truncatum* leaves, namely, kaempferol-3-*O*-*α*-L-rhamnoside, quercetin-3-*O*-*α*-L-rhamnopyranoside, kaempferol-3,7-di-*O*-*α*-L-rhamnoside, 1,2,3,4,6-penta-*O*-galloyl-*β*-D-glucopyranose (PGG). Compounds 1, 2, and 4 have been reported in *A. truncatum* leaves, compound **3** has been reported in this genus, but it is the first report in *A. truncatum* leaves.

It has been reported that compound **1** could significantly reduce the elevated inflammatory cell numbers in the bronchoalveolar lavage fluid (BALF) and lung tissues and inhibit the increase in Th2 cytokines in the lung and BALF, thus fully maintaining its anti-inflammatory and anti-asthmatic effects (Chung et al., 2015). As quercetin’s derivatives, compound **2** has been shown to have anti-inflammatory and anti-oxidant effects. For example, compound **2** could inhibit the TNF-alpha-stimulated production of cytokines and chemokines in HaCaT cells and attenuate the TNF-alpha-induced formation of inflammatory mediators and activation of the NF-kappa B and ERIC (Lee et al., 2013). In addition, compounds **1** and **2** also showed certain fatty acid synthase inhibitory activity, with IC50 of 45 and 50 μg/mL, respectively (Zhang et al., 2003). In the carrageenan-induced hind paw edema mice model, compound **3** also exhibited significant anti-inflammatory effects. At 50 mg/kg dose, compound **3** was shown to possess potent antinociceptive and anti-inflammatory activity without inducing any apparent acute toxicity as well as gastric damage (Toker et al., 2004). One of the polyphenolic compounds with strong anti-inflammatory, antioxidant, and anti-bacterial activities is compound **4**. It showed anti-inflammatory potential in monocytes via the modulation of cell activity and has been proved to have a broad-spectrum growth inhibitory effect on Gram-negative bacteria and Gram-positive bacteria. The MIC is between 16 and 32 μg/mL (Cho et al., 2010; Bobrowska et al., 2018). In addition, compound **4** is one of the main active components of *A. truncatum* leaves, which plays a key role in the FAS inhibitory activity and the growth inhibitory activity of various cancer cells (Zhao et al., 2014). To sum up, the compounds in *A. truncatum* leaves show various activities such as anti-inflammatory, anti-bacterial, anti-oxidant, hypoglycemic, and antitumor. The activities of these compounds can support the traditional usage of *A. truncatum* leaves, which is of great significance for the development and utilization of *A. truncatum* leaves (Figure 6).

### 4.3 Sustainable uses of *A. truncatum* resources

*A.r truncatum* is a tree species native to China and has a broad application prospect in the fields of oil production, gardening and environmental uses, ecological restoration, as well as new food and medicine development. The existing research has confirmed that *A. truncatum* has rich nutritional ingredients and medicinal values. Especially it has great potential in the treatment of Alzheimer’s disease and other ailments related to human wellbeing (Gu et al., 2019b). At present, however, the application of basic research and industrialization development of *A. truncatum* is not systematic and comprehensive, which greatly limits the development of relevant industries and the sustainable use of resources.

On one hand, the research on the extraction of active substances from *A. truncatum* and the processing of *A. truncatum* products are still in the laboratory stage, the development and factory survival of *A. truncatum* products need to be further promoted. On the other hand, the laboratory research of *A. truncatum* mainly focused on *A. truncatum* seed oil and flavonoids. The research work on other active ingredients is weak and less concerned. For example, the anti-inflammatory effect of *A. truncatum* leaves has not been reported before. This hinders people’s comprehensive understanding of this resource and limits the enthusiasm for industrial development. In addition, there are many seedling enterprises and individuals planting *A. truncatum*. But they lack scientific and standardized breeding techniques, which also influences the sustainable use of the *A. truncatum* resources.

Given the existing problems in the basic research and industrial development of *A. truncatum*, the high-quality *A. truncatum* varieties should be developed in the future, so as to efficiently obtain active ingredients. Then it will be possible to develop high-quality products, and realize the chain development of planting experiment, selection of excellent varieties and commodity development. Further studies will cover several areas, including those to strengthen the basic research on the application of *A. truncatum*, explore the biosynthesis and accumulation rules of active ingredients of *A. truncatum* through modern research technology. The result will provide strong theoretical basis for the efficient production of *A. truncatum* resources and the development of active ingredients. For instance, based on the chemical properties and pharmacological activities of *A. truncatum*, products of *A. truncatum* for neurological diseases, anti-cancer and skin-care will be developed and marketed.

## 5 Conclusion

Our work validated that ATLE has an inhibitory effect on SLS-induced apoptosis. Pretreatment of HaCaT cells with ATLE can downregulate the expression levels of inflammatory cytokines IL-6 and PGE2 in SLS-stimulated HaCaT cells. Four compounds were isolated from *A. truncatum* leaves, kaempferol-3-*O*-*α*-L-rhamnoside, quercetin-3-*O*-*α*-L-rhamnopyranoside, kaempferol-3,7-di-*O*-*α*-L-rhamnoside, and 1,2,3,4,6-Penta-*O*-galloyl-*β*-D-glucopyranose. These existing compounds have been proved to have significant anti-inflammatory activity, which may contribute to the anti-inflammatory effect of ATLE. Results presented in this study implied the protective effects of extracts from *A. truncatum* leaves on SLS-induced HaCaT cells. Therefore, our research may furnish scientific support for the traditional uses of *A. truncatum* leaves for treating itching and wounds from an anti-inflammation perspective. ATLE may be potential in future development for skin care.

## Data Availability

The raw data supporting the conclusion of this article will be made available by the authors, without undue reservation.
